# Downregulation of FeSOD-A expression in *Leishmania infantum* alters trivalent antimony and miltefosine susceptibility

**DOI:** 10.1186/s13071-021-04838-8

**Published:** 2021-07-15

**Authors:** Ana Maria Murta Santi, Paula Alves Silva, Isabella Fernandes Martins Santos, Silvane Maria Fonseca Murta

**Affiliations:** Grupo de Genômica Funcional de Parasitos (GFP), Instituto René Rachou, Fiocruz Minas, Avenida Augusto de Lima 1715, Belo Horizonte, MG CEP: 30190-002 Brazil

**Keywords:** *Leishmania infantum*, Iron superoxide dismutase, Knockout, Oxidative stress, Drug resistance

## Abstract

**Background:**

Superoxide dismutase (SOD), a central component of the antioxidant defence system of most organisms, removes excess superoxide anions by converting them to oxygen and hydrogen peroxide. As iron (Fe) SOD is absent in the human host, this enzyme is a promising molecular target for drug development against trypanosomatids.

**Results:**

We obtained *Leishmania infantum* mutant clones with lower FeSOD-A expression and investigated their phenotypes. Our attempts to delete this enzyme-coding gene using three different methodologies (conventional allelic replacement or two different CRISPR/methods) failed, as *FeSOD-A* gene copies were probably retained by aneuploidy or gene amplification. Promastigote forms of WT and mutant parasites were used in quantitative reverse-transcription polymerase chain reaction (RT-qPCR) and western blot analyses, and these parasite forms were also used to assess drug susceptibility. RT-qPCR and western blot analyses revealed that FeSOD-A transcript and protein levels were lower in *FeSOD-A*^*−/−/*+^ *L. infantum* mutant clones than in the wild-type (WT) parasite. The decrease in FeSOD-A expression in *L. infantum* did not interfere with the parasite growth or susceptibility to amphotericin B. Surprisingly, *FeSOD-A*^*−/−/*+^ *L. infantum* mutant clones were 1.5- to 2.0-fold more resistant to trivalent antimony and 2.4- to 2.7-fold more resistant to miltefosine. To investigate whether the decrease in FeSOD-A expression was compensated by other enzymes, the transcript levels of five FeSODs and six enzymes from the antioxidant defence system were assessed by RT-qPCR. The transcript level of the enzyme ascorbate peroxidase increased in both the *FeSOD-A*^*−/−/*+^ mutants tested. The *FeSOD-A*^*−/−/*+^ mutant parasites were 1.4- to 1.75-fold less tolerant to oxidative stress generated by menadione. Infection analysis using THP-1 macrophages showed that 72 h post-infection, the number of infected macrophages and their intracellular multiplication rate were lower in the *FeSOD-A*^*−/−/*+^ mutant clones than in the WT parasite.

**Conclusions:**

The unsuccessful attempts to delete *FeSOD-A* suggest that this gene is essential in *L. infantum*. This enzyme plays an important role in the defence against oxidative stress and infectivity in THP-1 macrophages. FeSOD-A-deficient *L. infantum* parasites deregulate their metabolic pathways related to antimony and miltefosine resistance.

**Graphic Abstract:**

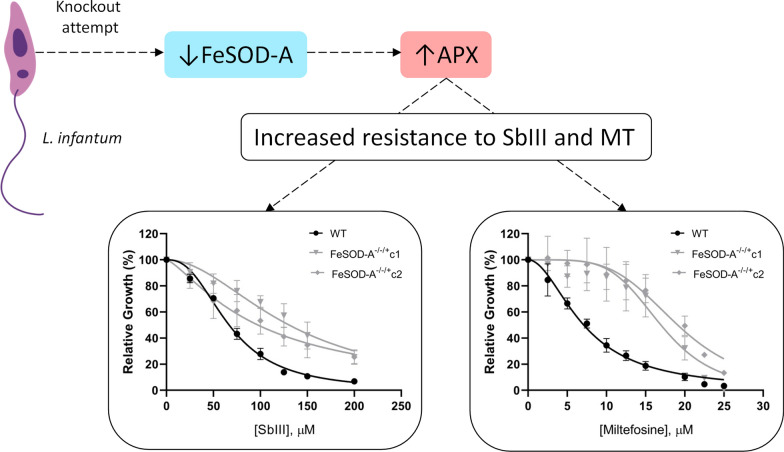

**Supplementary Information:**

The online version contains supplementary material available at 10.1186/s13071-021-04838-8.

## Background

Leishmaniases are zoonotic or anthroponotic infectious diseases caused by the protozoan parasites of the genus *Leishmania*. More than 21 species of *Leishmania* are known to infect humans and are transmitted by the bite of more than 90 species of female sandflies. Leishmaniasis mostly affects vulnerable populations and is detected in 98 countries across Europe, Africa, Asia, the Americas, and Oceania. More than 30,000 cases of visceral leishmaniasis and one million cases of cutaneous leishmaniasis are registered worldwide each year [1].

Only a few drugs are currently available for leishmaniasis treatment: pentavalent antimonials (Sb^V^), amphotericin B (AMB) and formulations, and miltefosine. The therapeutic failure of leishmaniasis is a major concern, and is probably related to the parasite’s resistance to treatment and the patient’s nutritional status, age, sex, and immunity. Another major concern with available therapies is their toxicity and side effects [2].

Given the present scenario, the development of new non-toxic and more effective drugs for the treatment of leishmaniasis is imperative. In this context, iron superoxide dismutase A (FeSOD-A) has garnered significant attention as a possible molecular target. It is an important enzyme in the antioxidant defence system that protects the parasites against superoxide radicals (O_2_^−^), which are converted to oxygen (O_2_) and hydrogen peroxide (H_2_O_2_) [3]. H_2_O_2_ is then metabolised by different enzymes with peroxidase activity, such as tryparedoxin peroxidase (TXNPx), ascorbate peroxidase (APX), peroxiredoxins (PRXs), and glutathione peroxidases (GPXs) [4].

The metalloenzyme SOD (EC 1.15.1.1) is a key component in the antioxidant defence system of most organisms and exhibits different metal cofactors at its active site [5]. In trypanosomatids, SODs have iron (Fe) in their structure and are classified as FeSOD-A expressed in the mitochondria [6, 7], FeSOD-B1 and FeSOD-B2 located in the glycosome [8], and FeSOD-C detected in the mitochondria [6]. As FeSOD is absent in the human host, this enzyme can serve as a promising molecular target for drug development against trypanosomatids.

FeSOD-deficient *L. tropica* was shown to be more sensitive to oxidative stress and FeSOD-deficient *L. donovani* had decreased ability to infect murine macrophages [9]. In addition, *L. amazonensis* deficient in FeSOD-A was found to be more sensitive to oxidative stress and less effective in producing lesions in mice [10]. Another study showed that a *L. donovani* strain isolated from a patient was resistant to miltefosine and overexpressed FeSOD-A [11]. *L. braziliensis* and *L. infantum* overexpressing FeSOD-A were more resistant to trivalent antimony and more tolerant to oxidative stress than their respective sensitive counterparts [12].

Considering the importance of this enzyme for the parasites and the potential for using FeSOD-A as a molecular target, here we investigate the impact of FeSOD-A downregulation in *L. infantum* on the drug susceptibility phenotype. We also evaluate tolerance to oxidative stress and infectivity by THP-1 macrophages, and determine the transcript levels of 11 other enzymes involved in the antioxidant defence system.

## Methods

*L. (Leishmania) infantum* RPV (MHOM/BR/2002/LPC-RPV) promastigotes were grown at 26 °C in M199 medium (Gibco) supplemented with 40 mM HEPES (pH 7.4), 5 μg/mL hemin, 2 μg/mL biopterin, 1 μg/mL biotin, 2 mM L-glutamine, 500 U/mL penicillin, 50 μg/mL streptomycin, and 10% inactivated fetal bovine serum [13]. Cultures were maintained by performing two weekly passages in which 1 × 10^6^ parasites were inoculated in 5 mL of medium. All experiments were performed using promastigotes in the logarithmic growth phase unless otherwise stated.

The *FeSOD-A* (LINF_080007900) knockout attempt was initially performed by two rounds of gene replacement by homologous recombination using neomycin phosphotransferase (NEO) and hygromycin phosphotransferase (HYG) as selectable markers [14]. The homology arms used for homologous recombination flank the FeSOD-A coding sequence and are 518 bp long in the 5′UTR and 459 bp long in the 3′UTR. Other knockout attempts were made using the CRISPR/Cas9 system.

The first attempt to knock out *FeSOD-A* using CRISPR was performed according to Zhang et al. [15]. The single-guide RNAs (sgRNAs) were selected using the Eukaryotic Pathogen CRISPR gRNA Design Tool (http://gRNA.ctegd.uga.edu), and the protospacer sequences were ligated into the pSPneoHHsgRNAaH vector that had been previously digested with the restriction enzyme *Bbs*I (New England Biolabs). Parasites containing the plasmid pLPhygCas9 were independently transfected with two different versions of the plasmid pSPneoHHsgRNAaH, containing sgRNAs 144 or 282, and a donor DNA to insert stop codons within the *FeSOD-A* coding sequence.

A second attempt to generate *FeSOD-A* null mutants using CRISPR/Cas9 was performed as previously described by Beneke et al. [16]. The plasmid pTB007, which has hygromycin as a resistance marker, was used to express SpCas9 and T7RNAP. Parasites carrying this plasmid and successfully expressing Cas9 were transfected with donor DNAs and templates for guide RNAs chosen with the LeishGEdit tool. Plasmids pTNeo v1 and pTBlast v1 were used for PCR amplification of the donors with 30 bp-long homology arms. sgRNA templates were generated by PCR using the G00 primer. All primers used to construct the plasmids and DNA fragments are listed in Additional file 1: Table S1.

All transfections were performed as previously described [14]. The selection of *Leishmania* clones was done by plating the parasites in semi-solid M199 medium, and selective drugs were added as per the resistance markers carried by the mutants as follows: 40 µg/mL G418 (Gibco), 400 µg/mL hygromycin B (Invitrogen), or 10 µg/mL blasticidin (Gibco). After that, selection drugs were used in the weekly passages of the cultures, but all experiments to assess the parasites' phenotype were conducted in the absence of selection drugs.

Deletion assessment was performed by PCR using primers to amplify the *FeSOD-A* coding sequence and primers to evaluate the replacement of *FeSOD-A* alleles by resistance marker sequences (Additional file 1: Table S1). Protein levels were assessed by western blotting using the polyclonal antibody anti-FeSOD-A [17] at a dilution of 1:500. Densitometric analysis was performed to compare FeSOD-A levels with the α-tubulin reference.

To evaluate the growth of the parasite, 1 × 10^6^ promastigotes/mL were inoculated in M199 medium, and the parasite concentration was determined daily using the Z1 Coulter Counter (Beckman Coulter) cell counter.

To evaluate the parasite’s susceptibility to antimony, AMB, miltefosine, and menadione, 2 × 10^6^ promastigotes were incubated in 1 mL M199 medium containing various concentrations of drugs. The number of parasites grown in the absence and presence of the drug after 48 h of incubation was determined using the Z1 Coulter Counter (Beckman Coulter). The 50% growth inhibitory concentration (IC_50_) was determined using the non-linear regression–variable slope model as per the equation "log (inhibitor) vs. response" in GraphPad Prism v.8.2.0.

To assess transcript levels, quantitative reverse-transcription PCR (RT-qPCR) analysis was performed using the cDNA of wild-type (WT) parasites and mutants. Promastigotes (approximately 10^8^ cells) were harvested and resuspended in 1 mL TRIzol Reagent (Invitrogen), and total RNA was extracted using the chloroform method. The RNA was treated with DNase I (Ambion), and the cDNA was obtained using Superscript II reverse transcriptase (Invitrogen) according to the manufacturer’s instructions. All cDNA samples were diluted to 100 ng/μL and used in the RT-qPCR amplification reaction performed using 1X SYBR GREEN master mix (Applied Biosystems) and the specific primers listed in Additional file 1: Table S1. Specific primers for each enzyme were designed using conserved nucleotide regions to amplify the different gene copies found in TritrypDB (tritrypdb.org). The levels of transcripts of the following enzymes were evaluated: six FeSODs (SODA [LINF_080007900], putative SODB1 [LINF_320024000], SODB2 [LINF_320024100], SOD putative [LINF_300033000], SOD putative [LINF_320033200], and SOD putative [LINF_340012900]) and six enzymes from the antioxidant defence system (APX [LINF_340005600], NADH-dependent fumarate reductase [FRD; LINF_350013000, LINF_350016600, and LINF_350016700], TXNPx [LINF_150018600, LINF_150018800, and LINF_150019000], type II GPX-like TXNPx [LINF_260013100], GPX putative [LINF_360038100], and PRX [LINF_230005400]). The DNA polymerase gene (LINF_160021500) was used as a constitutive normaliser. Amplifications were performed using a QuantStudio™ 12 K Flex system (Thermo Fisher Scientific). The PCR conditions were as follows: initial denaturation step at 95 °C for 5 min, followed by 40 cycles of denaturation at 95 °C for 15 s, annealing at 60 °C for 15 s, and extension at 60 °C for 30 s. Fluorescence was measured after each cycle. Transcript levels were determined using the comparative C_T_ method (2^−ΔΔCT^ method).

Cells derived from the human monocytic strain THP-1 were cultured in complete Rowell Park Memorial Institute (RPMI)-1640 medium (supplemented with 10% fetal bovine serum, 2 mM glutamine, 100 U/mL penicillin, and 100 µg/mL streptomycin). Monocytes were differentiated into macrophages by the addition of 20 ng/mL phorbol myristate acetate (PMA). After 72 h, the macrophages were infected with *Leishmania* cultures on the second day of the stationary phase (10 parasites per macrophage) for 6 h. The parasites that failed to infect the macrophages were washed away, and the infected macrophages were incubated for 72 h in RPMI-1640 medium. The infectivity of mutant clones was assessed immediately after the conclusion of the 6 h incubation period as well as after 72 h. The slides were stained with rapid panoptic (Laborclin) and photographed, and the infection was quantified by counting intracellular amastigotes using ImageJ free software.

### Statistical analysis

For all experiments, at least three technical replicates were performed for each of the three biological replicates. Data were analysed using GraphPad Prism v.8.2.0. Statistical significance was set at *p* < 0.05. The *p*-values were reported as per the GraphPad Prism format, where ns (*p* > 0.05), * (*p* ≤ 0.05), ** (*p* ≤ 0.01), *** (*p* ≤ 0.001), and **** (*p* ≤ 0.0001).

## Results

**Fig. 1:**
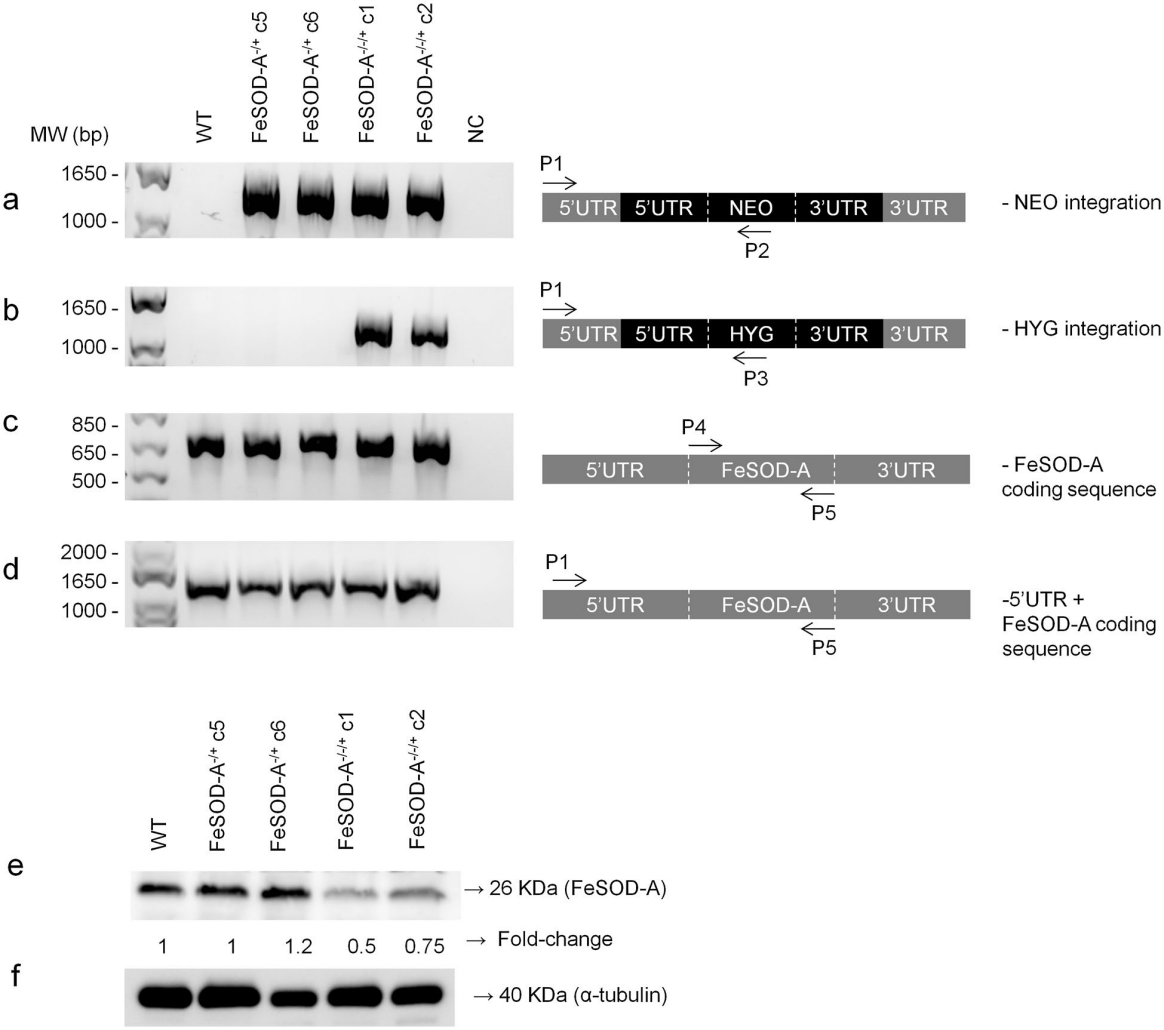
Attempt to knock out *FeSOD-A* using the conventional method of gene replacement by homologous recombination. First, the knockout was evaluated by PCR, using genomic DNA of wild-type parasites and mutants, *FeSOD-A*^−/+^ and *FeSOD-A*^−/−/+^. The correct integration of the resistance markers **a** NEO and **b** HYG was evaluated by PCR by annealing a primer in a 3′UTR region adjacent to the cassette (primer P1) and by another primer annealing within each resistance marker sequence (primers P2 or P3). The P1 primer is located 758 bp upstream of the *FeSOD-A* coding sequence, since the 3′UTR homology arm is 518 bp long. The cassettes used for homologous recombination are coloured in black. **c** Amplification of the *FeSOD-A* coding sequence by PCR using primers P4 and P5. **d** Amplification of the *FeSOD-A* coding sequence plus its 3′UTR by PCR using primers P1 and P5. **e** Western blot analysis of FeSOD-A expression in wild-type parasites and mutants (*FeSOD-A*^−/+^ and *FeSOD-A*^−/−/+^) using the anti-TcFeSOD-A polyclonal antibody that recognises a 26 kDa polypeptide. **f** Western blot analysis of α-tubulin using a monoclonal antibody, performed as a normaliser for the protein loading. MW: molecular weight standard; bp, base pairs; NC: negative control; WT: wild-type; KDa, kilodalton

The first attempt to knock out *FeSOD-A* in *L. infantum* was performed using two rounds of gene replacement by homologous recombination. This attempt was unsuccessful, leading to the retention of one copy of the gene by aneuploidy or gene amplification even after the correct integration of two different selectable markers replacing *FeSOD-A*. The presence of both NEO and HYG cassettes and their correct integration into the *L. infantum* genome replacing *FeSOD-A* was confirmed by PCR in all tested mutant clones (Fig. 1a, b). PCR was also used to verify the deletion of *FeSOD-A* in these parasites, and the results demonstrated its retention in the mutant parasites. The obtained clones were named *FeSOD-A*^*−/−/*+^ (Fig. 1c). Similarly, the attempts to knock out the *FeSOD-A* gene by CRISPR were unsuccessful. In all attempts, copies of the gene were retained by aneuploidy or gene amplification (Additional file 2: Figure S1 and Additional file 3: Figure S2).

FeSOD-A protein expression was evaluated in WT parasites, *FeSOD-A*^−/+^ clones C5 and C6, and *FeSOD-A*^−/−/+^ clones C1 and C2 by western blotting using a polyclonal antibody produced against the recombinant FeSOD-A protein. This antibody recognised a 26 kDa polypeptide in all the tested parasites. To normalise expression, FeSOD-A signals were compared to the signal of the α-tubulin by densitometric analysis. The WT parasites and *FeSOD-A*^−/+^ clone C5 had the same levels of FeSOD-A protein, but *FeSOD-A*^−/+^ clone 6 had a 20% increase in FeSOD-A expression when compared to the WT (Fig. 1e). On the other hand, the *FeSOD-A*^*−/−/*+^ mutant parasites showed lower levels of FeSOD-A than the WT parasite, as evident from a 50% decrease in clone 1 and a 25% decrease in clone 2.

The growth of promastigote forms of WT, *FeSOD-A*^−/+^, and *FeSOD-A*^−/−/+^ mutant clones was followed by daily parasite counting. No growth differences were observed between WT and mutant parasites (Additional file 4: Figure S3).

**Fig. 2:**
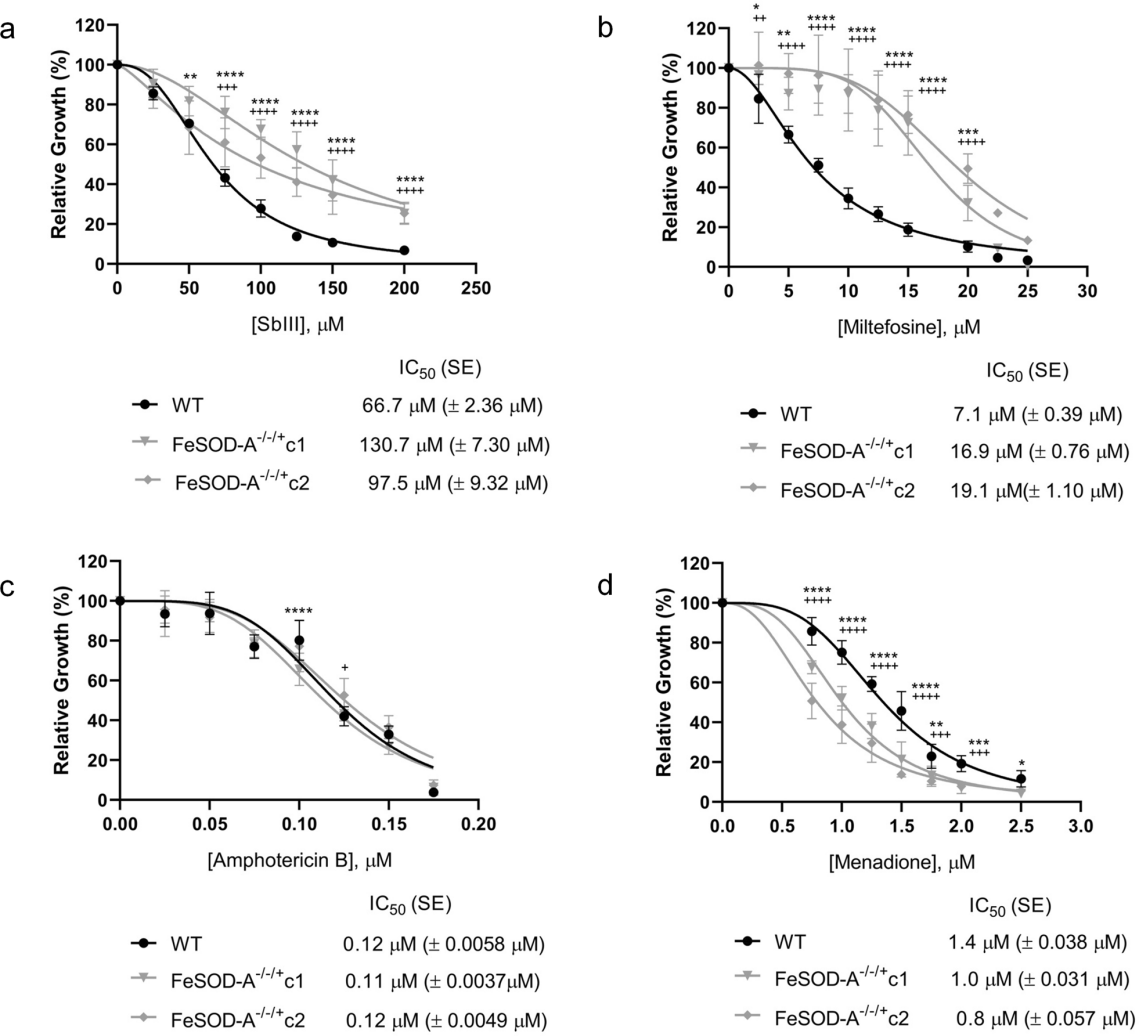
Drug susceptibility. WT and *FeSOD-A*^−/−/+^ parasites were cultured in the presence of different concentrations of **a** Sb^III^ (25–200 μM), **b** miltefosine (2.5–25 μM), **c** amphotericin B (0.025–0.175 μM), and **d** menadione (0.75–2.5 μM). Their growth was determined after 48 h of incubation with or without the drug. Data plotted in the dose–response curve represent the mean with standard deviations of three independent experiments performed in triplicate. The IC_50_ was determined through the non-linear regression–variable slope model, using the "log (inhibitor) vs. response" equation in GraphPad Prism v.8.2.0. A two-way ANOVA test with Bonferroni post hoc test was used to compare WT parasites and mutants for each drug concentration. * represents significant differences between the WT and the *FeSOD-A*^−/−/+^ clone C1 (* *p* < 0.05; ** *p* < 0.01; *** *p* < 0.001; **** *p* ≤ 0.0001). ^+^ represents significant differences between the WT and the *FeSOD-A*^−/−/+^ clone C2 (^+^ *p* < 0.05; ^++^ *p* < 0.01; ^+++^ *p* < 0.001; ^++++^ *p* ≤ 0.0001). Pairwise comparisons can be found in Additional File 6: Table S3

We investigated whether lower FeSOD-A expression alters the susceptibility of parasites to leishmanicidal drugs such as trivalent antimony (Sb^III^), miltefosine, and AMB. *FeSOD-A*^*−/−/*+^ mutants were found to be more resistant to Sb^III^ and miltefosine than the WT parasite. In the case of Sb^III^, the WT parasite presented an IC_50_ value of 66.7 µM and *FeSOD-A*^*−/−/*+^ clones C1 and C2 had IC_50_ values of 130.7 and 97.5 µM, respectively, which were 2.0- and 1.5-fold higher than the WT IC_50_ (Fig. 2a). We also evaluated the susceptibility to miltefosine and found an IC_50_ value of 7.1 µM for the WT parasite and 16.9 and 19.1 µM for *FeSOD-A*^*−/−/*+^ clones C1 and C2, respectively, indicating 2.4- and 2.7-fold higher values than the WT IC_50_ (Fig. 2b). However, WT and *FeSOD-A*^*−/−/*+^ clones C1 and C2 showed the same susceptibility to AMB, with their IC_50_ values ranging from 0.11 to 0.12 µM (Fig. 2c).

**Fig. 3:**
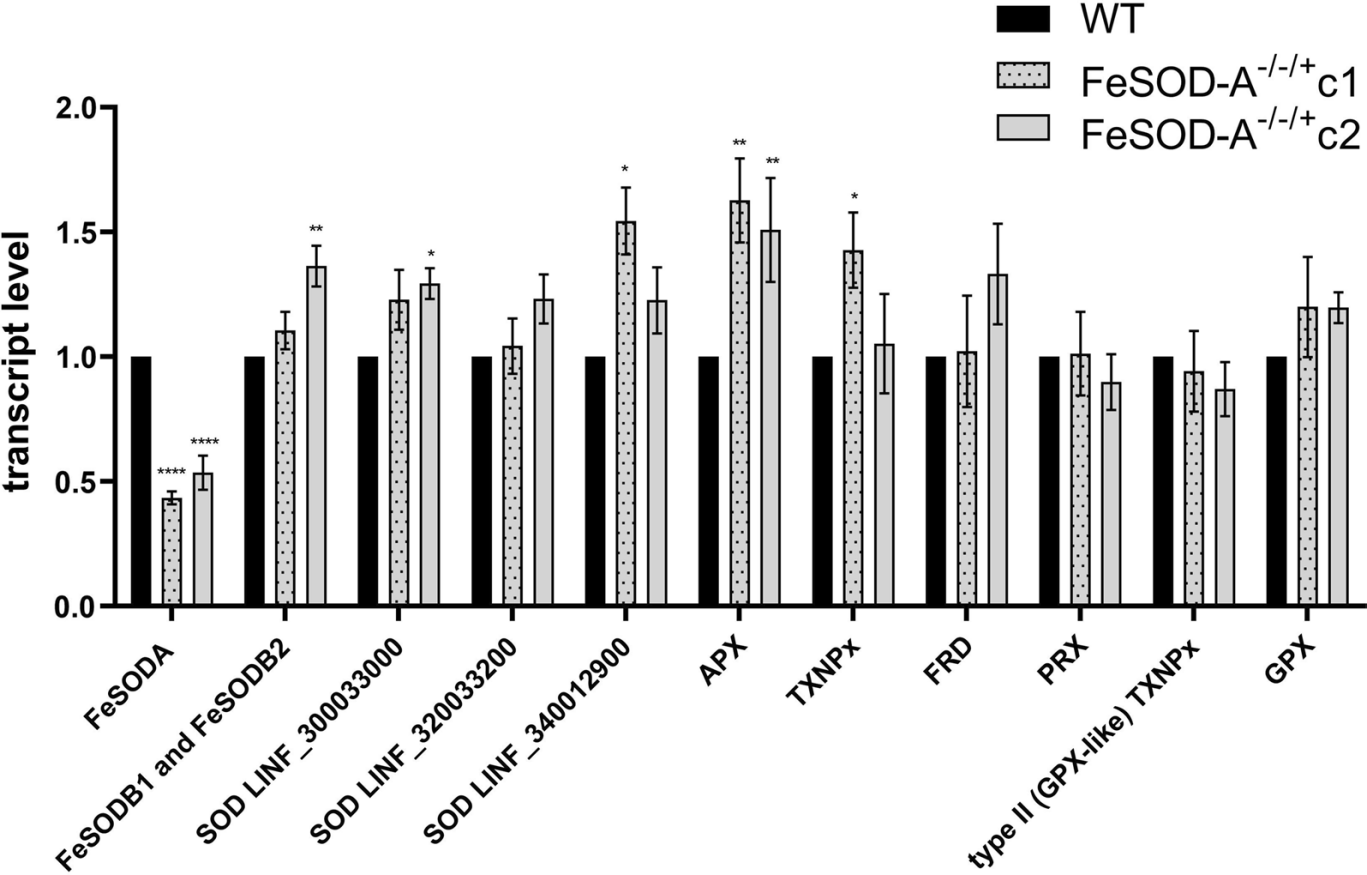
Transcription levels of superoxides, peroxidases, and fumarate reductase were assessed by RT-qPCR in wild-type parasites and *FeSOD-A*^−/−/+^ mutants. DNA polymerase gene (LINF_160021500) was used as constitutive normaliser, and the fold change was calculated by the 2^–∆∆Ct^ method. Enzymes analysed: iron superoxide dismutase SODA (LINF_080007900—FeSOD-A), iron superoxide dismutase – putative SODB1 (LINF_320024000—FeSOD-B1), iron superoxide dismutase – putative SODB2 (LINF_320024100—FeSOD-B2), superoxide dismutase—putative (SOD LINF_300033000), superoxide dismutase—putative (SOD LINF_320033200) and superoxide dismutase—putative (SOD LINF_340012900), ascorbate peroxidase (LINF_340005600—APX), tryparedoxin peroxidase (LINF_150018600, LINF_150018800, and LINF_150019000—TXNPx), NADH-dependent fumarate reductase (LINF_350013000, LINF_350016600, and LINF_350016700 FRD), peroxiredoxin (LINF_230005400—PRX), type II (glutathione peroxidase-like) tryparedoxin peroxidase (LINF_260013100—type II (GPX-like) TXNPx), glutathione peroxidase—putative (LINF_360038100—GPX). Ordinary one-way ANOVA test with Bonferroni post hoc test was used to compare WT parasites and mutants for each gene separately. * represents significant differences in relation to the wild-type parasite (* *p* < 0.05; ** *p* < 0.01; *** *p* < 0.001; **** *p* ≤ 0.0001). Pairwise comparisons: FeSODA WT vs. C1 *F*(2, 19) = 57.84, *p* < 0.0001; FeSODA WT vs. C2 *F*(2, 19) = 57.84, *p* < 0.0001; FeSODB1 and FeSODB2 WT vs. C2 *F*(2, 21) = 8.596, *p* = 0.0012; SOD LINF_300033000 WT vs. C2 *F*(2, 17) = 5.314; *p* = 0.0147; SOD LINF_340012900 WT vs. C1 *F*(2, 13) = 4.993, *p* = 0.0152; APX WT vs. C1 *F*(2, 11) = 14.44, *p* = 0.0013; APX WT vs. C2 *F*(2, 11) = 14.44, *p* = 0.0058; TXNPx WT vs. C1 *F*(2, 13) = 4.463, *p* = 0.0241

The results of RT-qPCR analyses were in line with the western blot findings and showed that *FeSOD-A* (LINF_080007900) transcript levels were 56% and 46% lower in *FeSOD-A*^*−/−/*+^ *L. infantum* mutant clones C1 and C2, respectively, than in the WT parasite (Fig. 3). To investigate whether the decrease in FeSOD-A expression was compensated by an increase in some other enzymes, the transcript levels of five FeSODs (FeSOD-B1, FeSOD-B2, and three SOD putative LINF_300033000, LINF_320033200, and LINF_340012900) and six other enzymes from the antioxidant defence system (APX, TXNPx, PRX, GPX, FDR, and type II [GPX-like] TXNPx) were assessed by RT-qPCR. The data demonstrated an increase of 63% and 51% in APX transcript levels in *L. infantum* mutant clones C1 and C2 as compared with that in the WT parasite, respectively. In addition, *FeSOD-A*^*−/−/*+^ clone C1 showed an increase in TXNPx and SOD putative (LINF_340012900) expression. *FeSOD-A*^*−/−/*+^ clone C2 had an increase in FeSOD putative SODB1 and SODB2 and SOD putative (LINF_300033000) expression (Fig. 3).

To further evaluate the mutant phenotype, WT and *FeSOD-A*^*−/−/*+^ mutant *L. infantum* parasites were incubated with increasing concentrations of menadione to investigate the effect of allelic replacement of *FeSOD-A* on protection against oxidative stress. Figure 2d shows the percentage of parasites grown at different concentrations of menadione after 48 h. The mutant parasites were 1.4- and 1.75-fold less tolerant to menadione than the WT parasite. While the WT parasite presented an IC_50_ value of 1.4 µM, the *FeSOD-A*^*−/−/*+^ mutant clones C1 and C2 presented IC_50_ values of 1.0 and 0.8 µM, respectively.

**Fig. 4:**
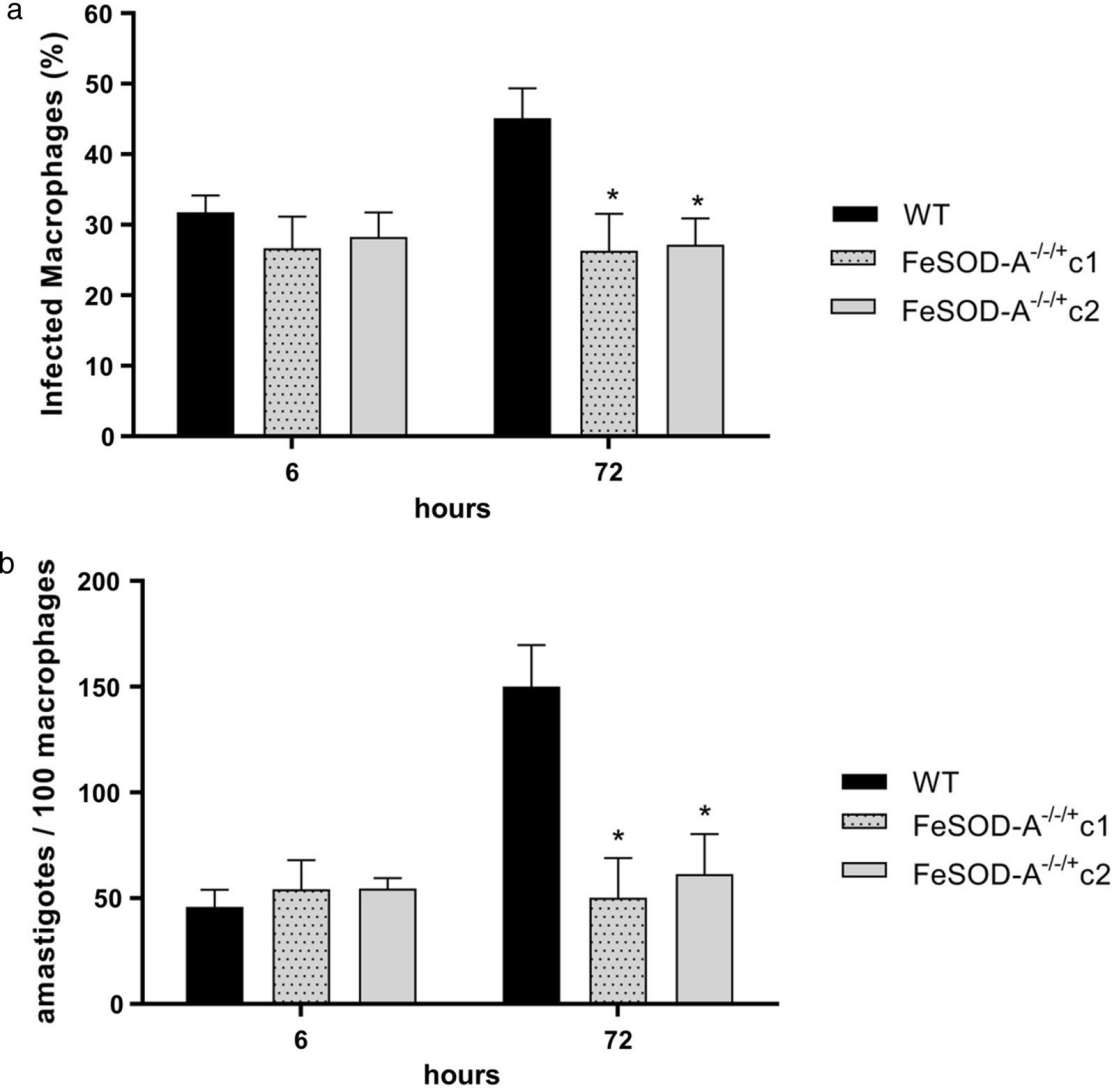
Analysis of the infectivity of *FeSOD-A*^−/−/+^ mutants in THP-1 macrophages. Macrophages were infected with wild-type parasites and with clones* FeSOD-A*^−/−/+^ in a ratio of 1:10 (10 parasites for each macrophage). Ordinary one-way ANOVA test with Bonferroni post hoc test was used to compare WT parasites and mutants at each time point; * represents significant differences in relation to the wild parasite (* *p* < 0.05; ** *p* < 0.01; *** *p* < 0.001; **** *p* ≤ 0.0001). **a** Percentage of infected macrophages, 6 and 72 h after infection. At 72 h—WT vs. C1 *F*(2, 8) = 6.270; *p* = 0.0351; at 72 h—WT vs. C2 *F*(2, 8) = 6.270; *p* = 0.0306. **b** Number of intracellular amastigotes per 100 macrophages, 6 and 72 h after infection. At 72 h—WT vs. C1 *F*(2, 7) = 8.364; *p* = 0.0164; at 72 h—WT vs. C2 *F*(2, 7) = 8.364; *p* = 0.0286

To evaluate whether the decrease in FeSOD-A expression changes the fitness of intracellular amastigote forms, we performed experimental infection of human macrophages THP-1 with WT and *FeSOD-A*^*−/−/*+^ mutant parasites. No difference in the infectivity of macrophages or intracellular multiplication rate was observed between the WT and *FeSOD-A*^*−/−/*+^ clones C1 and C2 mutant parasites 6 h after infection (Fig. 4). However, *FeSOD-A*^*−/−/*+^ mutants showed a lower number of infected macrophages (Fig. 4a), and fewer intracellular amastigotes were observed 72 h after infection (Fig. 4b).

## Discussion

In this study, we attempted to perform FeSOD-A knockout in *L. infantum* using three different methodologies (conventional allelic replacement and two CRISPR/Cas9 methods), but the copies of the gene were always retained, probably by aneuploidy or gene amplification [18]. The inability to delete FeSOD-A using three different methods suggests the essential role of this gene in *L. infantum*. It was recently demonstrated that *FeSOD-A* could not be deleted in *L. amazonensis* [10].

Several studies have shown that FeSOD-A is related to Sb^III^ and miltefosine susceptibility. Tessarollo et al. [12] reported higher activity of FeSOD enzymes in *L. infantum* and *L. braziliensis* resistant to Sb^III^. The authors also observed that *L. infantum* and *L. braziliensis* become more resistant to Sb^III^ following FeSOD-A overexpression [12]. Getachew and Gedamu [19] demonstrated that *L. donovani* overexpressing FeSOD-A was more resistant to miltefosine [19]. Another study reported that a miltefosine-resistant *L. donovani* isolate overexpressed FeSOD-A and demonstrated increased enzyme activity as compared to the susceptible isolate [11].

To our knowledge, this is the first study to assess the susceptibility of *FeSOD-A*^−/−/+^ mutant parasites to leishmanicidal drugs. Surprisingly, the *FeSOD-A*^*−/−/*+^ *L. infantum* mutant clones obtained in the present study were more resistant to both Sb^III^ and miltefosine than the WT. What may seem like a paradoxical result actually demonstrates the deregulation in the oxidative stress defence pathways and the ability of the parasite likely to compensate for the lower FeSOD-A expression. Interestingly, the existence of FeSOD-C in trypanosomatids was demonstrated in a study conducted by Dufernez et al. [6]. These authors showed that FeSOD-C, like FeSOD-A, is located in the mitochondria. MitoProt II analysis indicated that the protein encoded by the LINF_300033000 gene is very likely to be exported to the mitochondria (Additional file 5: Table S2), suggesting that LINF_300033000 would be FeSOD-C in *L. infantum*. Thus, we initially hypothesised that this enzyme could compensate for the role of FeSOD-A. However, despite the presence of FeSOD-C in the mitochondria of the parasites, it was impossible to knock out FeSOD-A expression. Furthermore, a significant increase in FeSOD-C transcript levels was detected only in FeSOD-A^*−/−/*+^ clone C2, which also had an increase in FeSOD putative SODB1 and SODB2 expression. In contrast, FeSOD-A^*−/−/*+^ clone C1 showed an increase in SOD putative (LINF_340012900) and TXNPx expression. The differing responses of the clones to the decrease in FeSOD-A expression suggest that the process of retention of the *FeSOD-A* gene copy by aneuploidy or gene amplification occurred in different ways between them. This heterogeneity is normal because *Leishmania* relies on aneuploidy as an adaptation mechanism [20]. Moreover, single-cell sequencing has confirmed the presence of different karyotypes within the same *Leishmania* clone [21, 22].

Among all evaluated enzymes, only APX transcript levels increased in both *FeSOD-A*^*−/−/*+^ clones. This redox enzyme of the trypanothione pathway converts H_2_O_2_ into water molecules, regulating oxidative stress in *Leishmania* [23]*.* Thus, an increase in APX transcript levels may favour the resistance of *FeSOD-A*^*−/−/*+^ mutant clones to Sb^III^ and miltefosine. Notably, *L. braziliensis* overexpressing APX was found to be eight times as resistant to Sb^III^ and 1.8 times as resistant to H_2_O_2_ as the WT parasite [24].

Our results show that FeSOD-A downregulation in *L. infantum* did not interfere with the pathogen’s susceptibility to AMB. Some factors can contribute to resistance to AMB, such as the lower drug binding to the membrane, owing to the changes in the sterol profile and membrane fluidity, AMB efflux, and free radical scavenger activity [25, 26]. However, downregulation of only one enzyme of the antioxidant defence system may not have a greater impact on resistance to this drug, as demonstrated in our results.

Here, we show that *FeSOD-A*^*−/−/*+^ mutant *L. infantum* parasites were more susceptible to oxidative stress generated by menadione than the WT parasite. This result is consistent with previous studies that demonstrated the role of FeSOD-A in protection against oxidative stress in *L. tropica* and *L. donovani* [9]*, L. amazonensis* [10], and *L. infantum* and *L. braziliensis* [12]. Furthermore, it was demonstrated that reduced expression of FeSOD-A in *L. amazonensis* resulted in mitochondrial oxidative damage and failure in promastigote-to-amastigote axenic differentiation [10]. Moreover, the lower infectivity of *FeSOD-A*^*−/−/*+^ parasites observed in our study is in agreement with the fact that they are less able to cope with oxidative stress from the host than the WT parasite. This result corroborates the previous finding that *L. amazonensis* with lower levels of FeSOD-A was unable to replicate in macrophages and did not generate lesions in mice [10]. The same study also showed that ROS is needed for parasite infectivity, and production of H_2_O_2_ by FeSOD-A is crucial in this process [10].

Despite the higher resistance of the mutants to Sb^III^ and miltefosine, they were less tolerant to menadione, as expected. Menadione is included in the vitamin K class of compounds and generates oxidative stress by increasing peroxide and superoxide radical levels [27]. Therefore, this difference in phenotypes would be possibly owing to the different response of the mutants to drugs that act in multiple pathways, such as Sb^III^ and miltefosine, as compared to their response to a compound that specifically generates oxidative stress such as menadione.

Several other enzymes not investigated in this study may also be involved in the *FeSOD-A*^*−/−/*+^ mutant clone phenotypes, as different enzymes are known to be associated with the maintenance of redox states in different subcellular compartments [4]. We also emphasise that spontaneous dismutation of O_2_^−^ to H_2_O_2_ is rapid and that the catalysis of this reaction by SODs is important mainly to prevent O_2_^−^ from reacting with cellular targets [28].

It is also very important to consider that here we only evaluated the transcript levels of some enzymes from the antioxidant defence system, but it is known that the transcript levels do not exactly match the protein expression in trypanosomatids. *Leishmania* genes are constitutively transcribed, and the regulation of gene expression largely occurs at the post-transcriptional level through RNA processing, RNA stability, translation efficiency, and post-translational modifications [29]. In addition, if FeSOD-A^*−/−/*+^ mutant clones present other alterations in their genomes, such as small insertions or deletions (indels), the protein functions may be impaired or favoured. This may alter the phenotype of the parasite. Several interesting studies have demonstrated the association between specific mutations and drug resistance phenotypes. For example, mutations in the miltefosine transporter that imparts resistance to miltefosine and AMB [30–32], mutations in aquaglyceroporin that cause antimony resistance [33], and mutations in the calcium-dependent protein kinase (CDPK1) are linked to paromomycin and antimony resistance [34]. Thus, we believe that it is possible that the *FeSOD-A*^*−/−/*+^ clones present changes that are undetectable through the analysis of only transcript levels.

## Conclusions

Here, we demonstrate the importance of FeSOD-A in the maintenance of redox states in *L. infantum*. The downregulation of this enzyme caused the parasite to be more susceptible to oxidative stress and decreased its ability to maintain infection in macrophages. Furthermore, the decrease in FeSOD-A expression caused an imbalance in the pathways related to Sb^III^ and miltefosine resistance.

## Supplementary Information

**Additional file 1: Table S1.** List of primers used in this study.

**Additional file 2: Figure S1.** First attempt to knock out *FeSOD-A* using the CRISPR/Cas9 system. For this attempt, parasites bearing the pLPhygCas9 plasmid were transfected with the pSPneoHH*sgRNA*aH, containing *sgRNA*_144 or sgRNA_282, and with their respective donor DNA containing stop codons. Two other transfections were performed to provide more donor DNA for mutant parasites. The knockout was evaluated by PCR, using genomic DNA of wild-type parasites, of parasites expressing Cas9 and of the mutant parasites (after one, two or three transfections with the donor DNA). The correct integration of the stop codons was evaluated by PCR by annealing a primer within the stop codon sequence and another primer within the FeSOD-A sequence. Two different guides were evaluated, **a** sgRNA144 and **b** sgRNA282. **c** After three transfections, the FeSOD-A protein levels were evaluated by western blot. MW: Molecular Weight Standard; bp, base pairs; NC: negative control; WT: wild-type; KDa, kilodalton.

**Additional file 3: Figure S2.** Second attempt to knock out *FeSOD-A* using the CRISPR/Cas9 system. For this attempt, parasites bearing the pT007_Cas9_T7 plasmid were transfected with DNA templates for the in vivo production of the sgRNAs and also with donor DNAs for allelic replacement of the FeSOD-A gene. Two different transfections were performed, one in which the parasites received only donor DNAs containing the NEO resistance marker and the other in which the parasites received donor DNAs containing both NEO and BDS markers. The donor DNAs are coloured in black. The correct integration of the resistance markers **a** NEO and **b** BSD was evaluated by PCR by annealing a primer in a 3′UTR region adjacent to the cassette (primer P1) and by another primer annealing within each resistance marker sequence (primers P2 or P6). The p1 primer is located 758 bp upstream of the FeSOD-A coding sequence. **c** Amplification of the FeSOD-A coding sequence by PCR using primers P4 and P5. **d** FeSOD-A protein levels were evaluated by western blot, comparing the wild-type and mutants. MW: Molecular Weight Standard; bp, base pairs; NC: negative control; WT: wild-type; KDa, kilodalton.

**Additional file 4: Figure S3.** Growth of WT, *FeSOD-A*^−/+^ and *FeSOD-A*^−/−/+^ parasites. Initially, 1 × 10^6^ parasites per mL were inoculated in M199 medium. The parasites were cultivated and the growth was evaluated by daily counting of the parasites using the Z1 Coulter Counter. The data present the average of three independent experiments performed in triplicate, and the growth curves were built using a non-linear regression model with the “beta growth then decay” equation in GraphPad Prism v.8.2.0.

**Additional file 5: Table S2.** Probability to export FeSOD isoforms to mitochondria according to the results obtained at MitoProt II—v1.101.

**Additional file 6: Table S3.** Statistics of IC_50_ experiments. Two-way ANOVA dose vs. response—multiple comparisons.

## Data Availability

The datasets supporting the conclusions of this article are included within the article and its additional files.

## Abbreviations

AMB: Amphotericin B
APX: Ascorbate peroxidase
FeSOD: Iron superoxide dismutase
GPX: Glutathione peroxidases
HYG: Hygromycin phosphotransferase
NEO: Neomycin phosphotransferase
PRX: Peroxiredoxin
Sb^III^: Trivalent antimony
Sb^V^: Pentavalent antimony
TXNPx: Tryparedoxin peroxidase
