# Supplementary material for: Downregulation of FeSOD-A expression in Leishmania infantum alters trivalent antimony and miltefosine susceptibility
Source: Parasit Vectors. 2021 Jul 15;14:366. doi: 10.1186/s13071-021-04838-8 (PMC8281622; doi:10.1186/s13071-021-04838-8)
Supplement: Supplementary file 1 — Additional file 1: Table S1. List of primers used in this study. [file 13071_2021_4838_MOESM1_ESM.docx]

| \| **Additional file 1: Table S1**. List of primers used in this study \| \| \| \| --- \| --- \| --- \| \| **PRIMER NAME** \| **PRIMER SEQUENCE 5’ → 3’** \| \| \| XbaI.5'*Li*SOD.Fw \| TCTAGAGTGTGTGGGCATGTG \| \| \| degHYG.5'*Li*SOD.Rev \| GTGAGTTCAGGCTTTTTCATTCGGAATGCGGGAGGACTGG \| \| \| degNEO.5'*Li*SOD.Rev \| AATCCATCTTGTTCAATCATTCGGAATGCGGGAGGACTGG \| \| \| NEO_Fw \| ATGATTGAACAAGATGGATTGCACGC \| \| \| deg3'LiSOD.NEO.Rev \| CAGATGCATGTACGACTCTCTCAGAAGAACTCGTCAAGAAG \| \| \| HYG.Fw \| ATGAAAAAGCCTGAACTCACCGCG \| \| \| deg3'*Li*SOD.HYG.Rev \| CAGATGCATGTACGACTCTCTCATCGATGATGGGGATCTG \| \| \| 3'*Li*SOD.Fw \| GAGAGTCGTACATGCATCTGTACC \| \| \| SalI.3'*Li*SOD.rev \| GTCGACAAAGAAACAGACGGCTA \| \| \| P1 (5'out) \| CGTGTACCGGTAGCGTAAT \| \| \| P2 (NEO_mid.RV) \| GCCAACGCTATGTCCTGATA \| \| \| P3 (HYG_mid.RV) \| CTGACGGTGTCGTCCATAAC \| \| \| P6 (BSD_mid.RV) \| CCATCACTGTCCTTCACTATCG \| \| \| P4 (LiFeSodA*.*Fw) \| ATGTTCCGCCGCGTCTC \| \| \| P5 (LiFeSodA.Rv) \| TTACTTCGTGGCCTTCTCATACATC \| \| \| Donor282Fw \| GGCAGCACTGAGAGCAAGGTCATGTTCAACTAGATAGATAGggatccCAG \| \| \| P8 (Donor282Rv) \| GAAGGAATGGTTGAAGTGCTGAGCGGCCTGggatccCTATCTATCTA \| \| \| Donor144Fw \| GGCATCCAGCCCGTCATGAGCTCCCGTCAGTAGATAGATAGggatccTTG \| \| \| P7 (Donor144Rv) \| GCTGTGGTGCTTCTTGTAGTGCAGCTCCAAggatccCTATCTATCTA \| \| \| [LiFeSodA_282_HH_Fw](http://grna.ctegd.uga.edu/jobs/jobs/jobs/jobs/63eLkDTvQtfMw9gBt08mToBDj0wHGj-FeSodA/gRNAclickinfo/FeSodA_75.html) \| GGTGGAGCAAGGTCATGTTCAACC \| \| \| [LiFeSodA_282_Rv](http://grna.ctegd.uga.edu/jobs/jobs/jobs/jobs/63eLkDTvQtfMw9gBt08mToBDj0wHGj-FeSodA/gRNAclickinfo/FeSodA_75.html) \| AAACGGTTGAACATGACCTTGCTC \| \| \| [LiFeSodA_144_HH_Fw](http://grna.ctegd.uga.edu/jobs/jobs/jobs/jobs/63eLkDTvQtfMw9gBt08mToBDj0wHGj-FeSodA/gRNAclickinfo/FeSodA_75.html) \| GGTGGCGTCATGAGCTCCCGTCAGT \| \| \| [LiFeSodA_144_Rv](http://grna.ctegd.uga.edu/jobs/jobs/jobs/jobs/63eLkDTvQtfMw9gBt08mToBDj0wHGj-FeSodA/gRNAclickinfo/FeSodA_75.html) \| AAACACTGACGGGAGCTCATGACGC \| \| \| Upstream forward primer \| TCGCACGACTCCACGCCTCCACTCTCTCCCgtataatgcagacctgctgc \| \| \| 5' sgRNA primer \| gaaattaatacgactcactataggACTGGAGTGCTTGGGGGAAAgttttagagctagaaatagc \| \| \| Downstream RV \| GGACGCGCGTGCATCGCTCGGTCCCTCCCTccaatttgagagacctgtgc \| \| \| 3' sgRNA primer \| gaaattaatacgactcactataggCCAATATGCTGAGGTGAGGAgttttagagctagaaatagc \| \| \| G00 gRNA scaffold \| Aaaagcaccgactcggtgccactttttcaagttgataacggactagccttattttaacttgctatttctagctctaaaac \| \| \| SOD_A_RTqPCR_FW \| GAGCACGCCTACTACAAAGA \| \| \| SOD_A_RTqPCR_RV \| CTTCGTGGCCTTCTCATACA \| \| \| SOD_B1/2_RTqPCR_FW \| TCACCTTCCACCACGAGAA \| \| \| SOD_B1/2_RTqPCR_RV \| CAGACTTGATGATGTCCACCAG \| \| \| SOD33000_RTqPCR_FW \| CTACTGCTTCTGGTATCGTTCTC \| \| \| SOD33000_RTqPCR_RV \| CAGCGTCTAAGGTCCCATTT \| \| \| SOD33200_RTqPCR_FW \| CTGGAGTTTCCGTGGTACAAG \| \| \| SOD33200_RTqPCR_RV \| GCCCTCGATGAGTTGGTTAAG \| \| \| SOD12900_RTqPCR_FW \| TCACAAACACTGCCTCTAACTC \| \| \| SOD12900_RTqPCR_RV \| CCCGTTGTTGTAGTGGTACTC \| \| \| Fumarate_RTqPCR_Fw \| GCTTCTCGAACGACCACAC \| \| \| Fumarate_RTqPCR_Rv \| GCAGCTGCACCTTGTCC \| \| \| Ascorbate_RTqPCR_FW \| CATCAAATTCTCCGGCTACCA \| \| \| Ascorbate_RTqPCR_RV \| TCTCAACCTTCGGATTCAAGAC \| \| \| tryparedoxin _RTqPCR_FW \| \| TCGCTTCAACGAGCTCAAC \| \| tryparedoxin _RTqPCR_RV \| \| TCGGCCAGCATTGGAATC \| \| DNApol_RTqPCR_ Fw \| \| CGAGGGCAAGACATAC \| \| DNApol_RTqPCR_ Rev \| \| GAGAGCGGGCACCAATCAC \| \| peroxidoxin_FW \| \| GCTCTTCGAGGGCTCTTTATC \| \| peroxidoxin_RV \| \| GCCCACAGGAAGGTCATTTA \| \| glutathione peroxidase_FW \| \| CGCAGTTTGCGAATCAAGAG \| \| glutathione peroxidase_RV \| \| CCGGTCAAAGACAGGAAAGA \| \| type II (glutathione peroxidase-like) tryparedoxin peroxidase_FW \| \| CCATCCGCTTCTCATCTACAA \| \| type II (glutathione peroxidase-like) tryparedoxin peroxidase_RV \| \| GAAGCCGAGATGCTTGTACT \| |  |
| --- | --- | --- | --- | --- | --- | --- | --- | --- | --- | --- | --- | --- | --- | --- | --- | --- | --- | --- | --- | --- | --- | --- | --- | --- | --- | --- | --- | --- | --- | --- | --- | --- | --- | --- | --- | --- | --- | --- | --- | --- | --- | --- | --- | --- | --- | --- | --- | --- | --- | --- | --- | --- | --- | --- | --- | --- | --- | --- | --- | --- | --- | --- | --- | --- | --- | --- | --- | --- | --- | --- | --- | --- | --- | --- | --- | --- | --- | --- | --- | --- | --- | --- | --- | --- | --- | --- | --- | --- | --- | --- | --- | --- | --- | --- | --- | --- | --- | --- | --- | --- | --- | --- | --- | --- | --- | --- | --- | --- | --- | --- | --- | --- | --- | --- | --- | --- | --- | --- | --- | --- | --- | --- | --- | --- | --- | --- | --- | --- | --- | --- | --- | --- | --- | --- | --- | --- | --- | --- | --- | --- | --- | --- | --- | --- | --- | --- | --- | --- | --- | --- | --- | --- | --- | --- | --- | --- | --- | --- | --- | --- | --- | --- | --- |
