# Supplementary figures and images for: Downregulation of FeSOD-A expression in Leishmania infantum alters trivalent antimony and miltefosine susceptibility
Source: Parasit Vectors. 2021 Jul 15;14:366. doi: 10.1186/s13071-021-04838-8 (PMC8281622; doi:10.1186/s13071-021-04838-8)

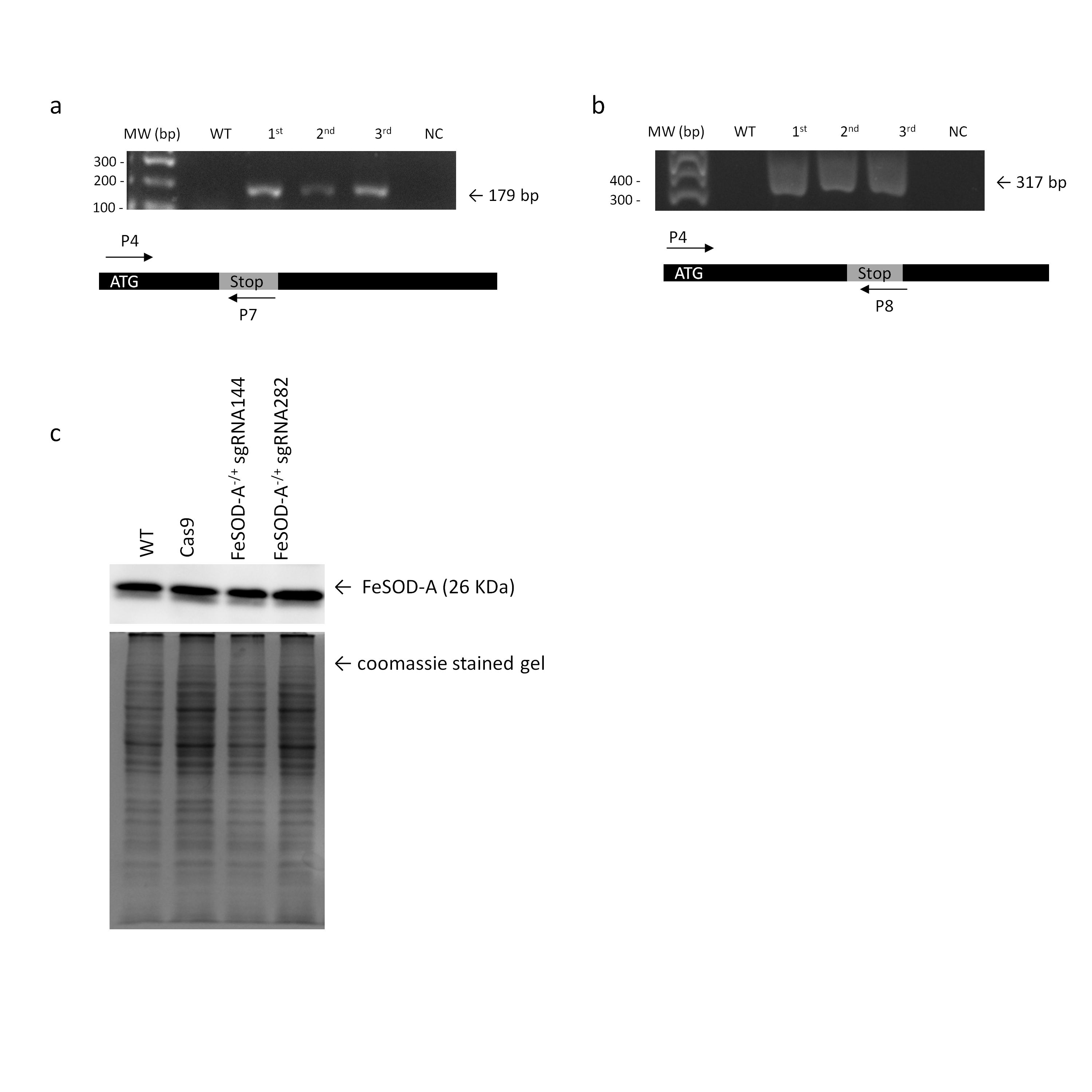

Supplement: Supplementary file 2 — Additional file 2: Figure S1. First attempt to knock out FeSOD-A using the CRISPR/Cas9 system. For this attempt, parasites bearing the pLPhygCas9 plasmid were transfected with the pSPneoHHsgRNAaH, containing sgRNA_144 or sgRNA_282, and with their respective donor DNA containing stop codons. Two other transfections were performed to provide more donor DNA for mutant parasites. The knockout was evaluated by PCR, using genomic DNA of wild-type parasites, of parasites expressing Cas9 and of the mutant parasites (after one, two or three transfections with the donor DNA). The correct integration of the stop codons was evaluated by PCR by annealing a primer within the stop codon sequence and another primer within the FeSOD-A sequence. Two different guides were evaluated, a sgRNA144 and b sgRNA282. c After three transfections, the FeSOD-A protein levels were evaluated by western blot. MW: Molecular Weight Standard; bp, base pairs; NC: negative control; WT: wild-type; KDa, kilodalton. [file 13071_2021_4838_MOESM2_ESM.png]

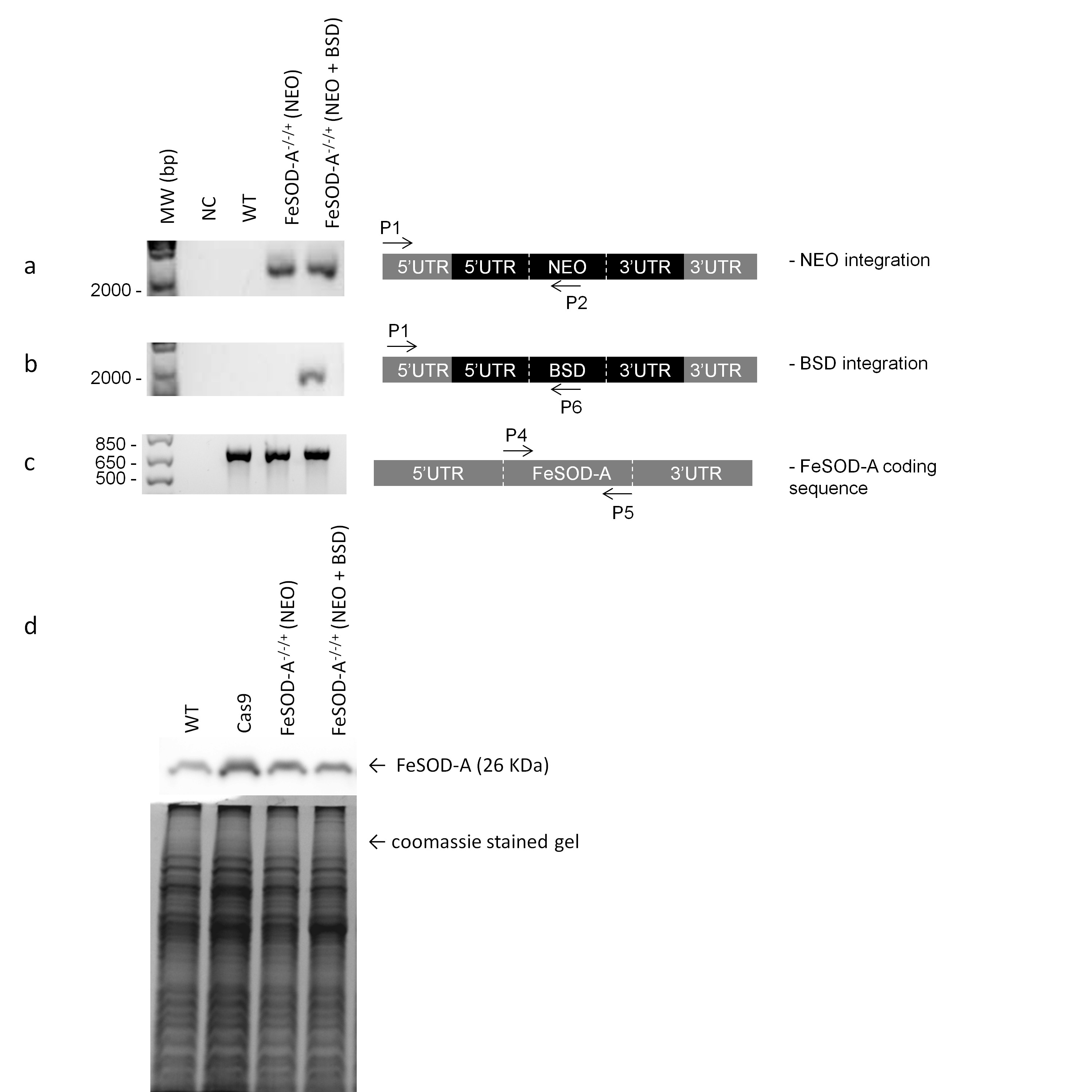

Supplement: Supplementary file 3 — Additional file 3: Figure S2. Second attempt to knock out FeSOD-A using the CRISPR/Cas9 system. For this attempt, parasites bearing the pT007_Cas9_T7 plasmid were transfected with DNA templates for the in vivo production of the sgRNAs and also with donor DNAs for allelic replacement of the FeSOD-A gene. Two different transfections were performed, one in which the parasites received only donor DNAs containing the NEO resistance marker and the other in which the parasites received donor DNAs containing both NEO and BDS markers. The donor DNAs are coloured in black. The correct integration of the resistance markers a NEO and b BSD was evaluated by PCR by annealing a primer in a 3′UTR region adjacent to the cassette (primer P1) and by another primer annealing within each resistance marker sequence (primers P2 or P6). The p1 primer is located 758 bp upstream of the FeSOD-A coding sequence. c Amplification of the FeSOD-A coding sequence by PCR using primers P4 and P5. d FeSOD-A protein levels were evaluated by western blot, comparing the wild-type and mutants. MW: Molecular Weight Standard; bp, base pairs; NC: negative control; WT: wild-type; KDa, kilodalton. [file 13071_2021_4838_MOESM3_ESM.png]

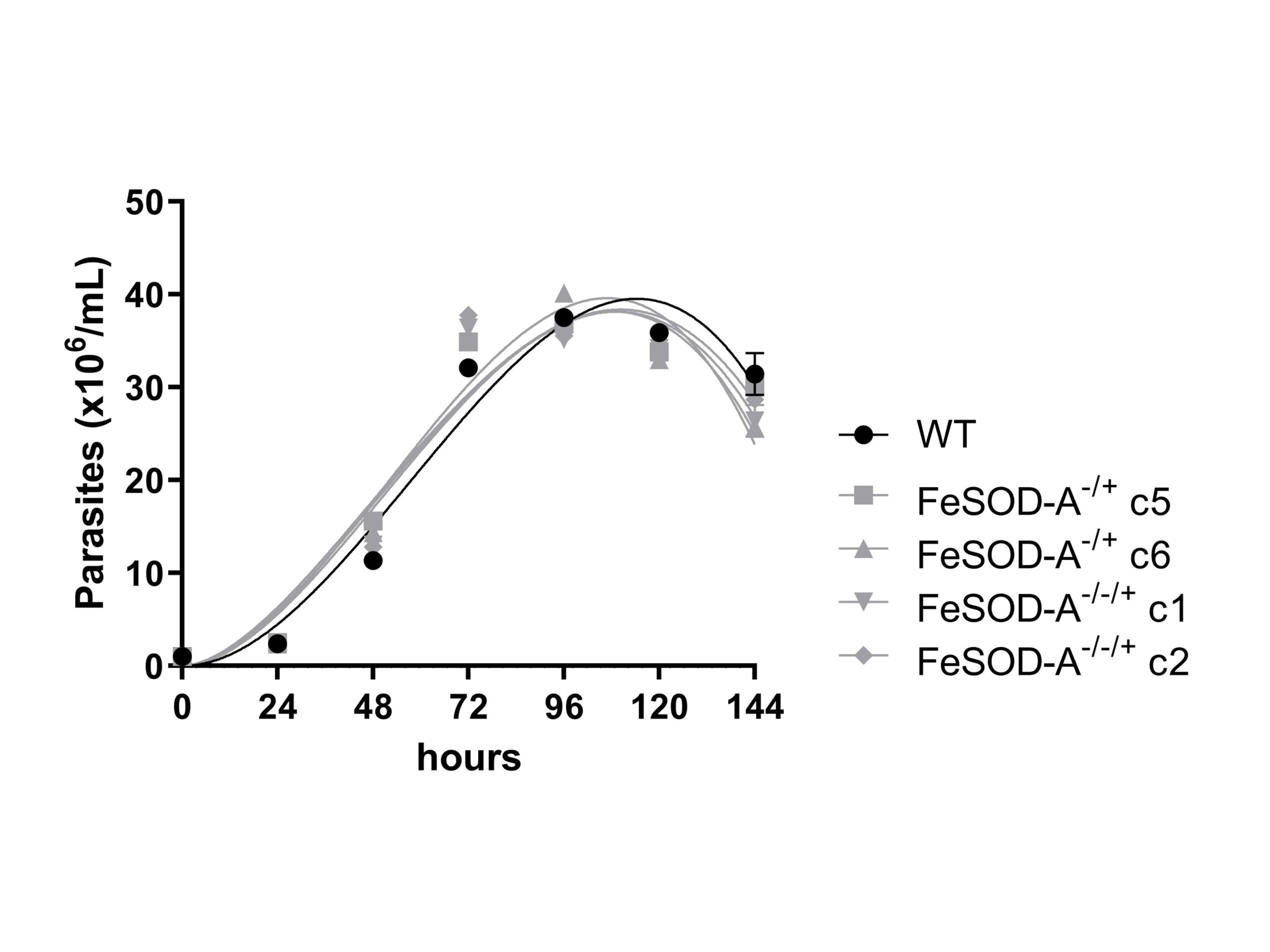

Supplement: Supplementary file 4 — Additional file 4: Figure S3. Growth of WT, FeSOD-A−/+ and FeSOD-A−/−/+ parasites. Initially, 1 × 10^6^ parasites per mL were inoculated in M199 medium. The parasites were cultivated and the growth was evaluated by daily counting of the parasites using the Z1 Coulter Counter. The data present the average of three independent experiments performed in triplicate, and the growth curves were built using a non-linear regression model with the “beta growth then decay” equation in GraphPad Prism v.8.2.0. [file 13071_2021_4838_MOESM4_ESM.png]
