# Supplementary material for: Downregulation of FeSOD-A expression in Leishmania infantum alters trivalent antimony and miltefosine susceptibility
Source: Parasit Vectors. 2021 Jul 15;14:366. doi: 10.1186/s13071-021-04838-8 (PMC8281622; doi:10.1186/s13071-021-04838-8)
Supplement: Supplementary file 5 — Additional file 5: Table S2. Probability to export FeSOD isoforms to mitochondria according to the results obtained at MitoProt II—v1.101. [file 13071_2021_4838_MOESM5_ESM.docx]

**Additional file 5: Table S2**. Probability to export FeSOD isoforms to mitochondria according to the results obtained at MitoProt II - v1.101

(Available at <https://ihg.helmholtz-muenchen.de/ihg/mitoprot.html>)

| **Gene ID (TriTrypDB)** | **Organism** | **Product Description** |  | **PROBABILITY of export to mitochondria (MitoProt)** |
| --- | --- | --- | --- | --- |
| [LINF_080007900](https://tritrypdb.org/tritrypdb/app/record/gene/LINF_080007900) | *L. infantum* JPCM5 | iron superoxide dismutase | SODA | **0.8921** |
| [LINF_300033000](https://tritrypdb.org/tritrypdb/app/record/gene/LINF_300033000) | *L. infantum* JPCM5 | superoxide dismutase - putative |  | **0.9595** |
| [LINF_320024000](https://tritrypdb.org/tritrypdb/app/record/gene/LINF_320024000) | *L. infantum* JPCM5 | iron superoxide dismutase - putative | SODB1 | **0.0468** |
| [LINF_320024100](https://tritrypdb.org/tritrypdb/app/record/gene/LINF_320024100) | *L. infantum* JPCM5 | iron superoxide dismutase - putative | SODB2 | **0.0565** |
| [LINF_320033200](https://tritrypdb.org/tritrypdb/app/record/gene/LINF_320033200) | *L. infantum* JPCM5 | superoxide dismutase - putative |  | **0.0621** |
| [LINF_340012900](https://tritrypdb.org/tritrypdb/app/record/gene/LINF_340012900) | *L. infantum* JPCM5 | hypothetical protein - conserved |  | **0.4270** |
