# Supplementary material for: Downregulation of FeSOD-A expression in Leishmania infantum alters trivalent antimony and miltefosine susceptibility
Source: Parasit Vectors. 2021 Jul 15;14:366. doi: 10.1186/s13071-021-04838-8 (PMC8281622; doi:10.1186/s13071-021-04838-8)
Supplement: Supplementary file 6 — Additional file 6: Table S3. Statistics of IC50 experiments. Two-way ANOVA dose vs. response—multiple comparisons. [file 13071_2021_4838_MOESM6_ESM.docx]

| **Additional file 6: Table S3**. Statistics of IC_50_ experiments. 2way ANOVA dose vs. response – Multiple Comparisons  **Sb^III^** | | | | | | | |
| --- | --- | --- | --- | --- | --- | --- | --- |
|  |  |  |  |  |  |  |  |
| **Bonferroni's multiple comparisons test** | **Mean Diff.** | **95.00% CI of diff.** | **Significant?** | **Summary** | **Adjusted P Value** | **t** | **DF** |
|  |  |  |  |  |  |  |  |
| 0 |  |  |  |  |  |  |  |
| WT vs. FeSOD-A^-/-/+^c1 | 0 | -13.56 to 13.56 | No | ns | >0.9999 | 0 | 123 |
| WT vs. FeSOD-A^-/-/+^c2 | 0 | -13.56 to 13.56 | No | ns | >0.9999 | 0 | 123 |
|  |  |  |  |  |  |  |  |
| 25 |  |  |  |  |  |  |  |
| WT vs. FeSOD-A^-/-/+^c1 | -4.864 | -14.10 to 4.373 | No | ns | 0.4689 | 1.195 | 123 |
| WT vs. FeSOD-A^-/-/+^c2 | 1.872 | -8.182 to 11.93 | No | ns | >0.9999 | 0.4226 | 123 |
|  |  |  |  |  |  |  |  |
| 50 |  |  |  |  |  |  |  |
| WT vs. FeSOD-A^-/-/+^c1 | -11.2 | -19.95 to -2.450 | Yes | ** | 0.0087 | 2.904 | 123 |
| WT vs. FeSOD-A^-/-/+^c2 | 2.078 | -7.508 to 11.66 | No | ns | >0.9999 | 0.4919 | 123 |
|  |  |  |  |  |  |  |  |
| 75 |  |  |  |  |  |  |  |
| WT vs. FeSOD-A^-/-/+^c1 | -32.79 | -42.05 to -23.53 | Yes | **** | <0.0001 | 8.035 | 123 |
| WT vs. FeSOD-A^-/-/+^c2 | -17.79 | -27.85 to -7.740 | Yes | *** | 0.0002 | 4.016 | 123 |
|  |  |  |  |  |  |  |  |
| 100 |  |  |  |  |  |  |  |
| WT vs. FeSOD-A^-/-/+^c1 | -39.73 | -50.23 to -29.23 | Yes | **** | <0.0001 | 8.585 | 123 |
| WT vs. FeSOD-A^-/-/+^c2 | -25.49 | -35.54 to -15.43 | Yes | **** | <0.0001 | 5.753 | 123 |
|  |  |  |  |  |  |  |  |
| 125 |  |  |  |  |  |  |  |
| WT vs. FeSOD-A^-/-/+^c1 | -43.4 | -52.15 to -34.65 | Yes | **** | <0.0001 | 11.25 | 123 |
| WT vs. FeSOD-A^-/-/+^c2 | -27.3 | -36.88 to -17.71 | Yes | **** | <0.0001 | 6.462 | 123 |
|  |  |  |  |  |  |  |  |
| 150 |  |  |  |  |  |  |  |
| WT vs. FeSOD-A^-/-/+^c1 | -31.2 | -39.95 to -22.45 | Yes | **** | <0.0001 | 8.091 | 123 |
| WT vs. FeSOD-A^-/-/+^c2 | -23.93 | -33.52 to -14.35 | Yes | **** | <0.0001 | 5.665 | 123 |
|  |  |  |  |  |  |  |  |
| 200 |  |  |  |  |  |  |  |
| WT vs. FeSOD-A^-/-/+^c1 | -18.43 | -27.18 to -9.678 | Yes | **** | <0.0001 | 4.779 | 123 |
| WT vs. FeSOD-A^-/-/+^c2 | -18.56 | -28.14 to -8.971 | Yes | **** | <0.0001 | 4.393 | 123 |

| **Miltefosine** |  |  |  |  |  |  |  |
| --- | --- | --- | --- | --- | --- | --- | --- |
|  |  |  |  |  |  |  |  |
| **Bonferroni's multiple comparisons test** | **Mean Diff.** | **95.00% CI of diff.** | **Significant?** | **Summary** | **Adjusted P Value** | **t** | **DF** |
|  |  |  |  |  |  |  |  |
| 0 |  |  |  |  |  |  |  |
| WT vs. FeSOD-A-/-/+c1 | 0 | -20.74 to 20.74 | No | ns | >0.9999 | 0 | 169 |
| WT vs. FeSOD-A-/-/+c2 | 0 | -20.74 to 20.74 | No | ns | >0.9999 | 0 | 169 |
|  |  |  |  |  |  |  |  |
| 2.5 |  |  |  |  |  |  |  |
| WT vs. FeSOD-A-/-/+c1 | -12.01 | -23.98 to -0.04037 | Yes | * | 0.049 | 2.269 | 169 |
| WT vs. FeSOD-A-/-/+c2 | -16.83 | -28.80 to -4.861 | Yes | ** | 0.0035 | 3.18 | 169 |
|  |  |  |  |  |  |  |  |
| 5 |  |  |  |  |  |  |  |
| WT vs. FeSOD-A-/-/+c1 | -20.57 | -33.95 to -7.182 | Yes | ** | 0.0013 | 3.475 | 169 |
| WT vs. FeSOD-A-/-/+c2 | -30.72 | -44.10 to -17.33 | Yes | **** | <0.0001 | 5.19 | 169 |
|  |  |  |  |  |  |  |  |
| 7.5 |  |  |  |  |  |  |  |
| WT vs. FeSOD-A-/-/+c1 | -38.12 | -51.51 to -24.74 | Yes | **** | <0.0001 | 6.441 | 169 |
| WT vs. FeSOD-A-/-/+c2 | -45.33 | -58.71 to -31.94 | Yes | **** | <0.0001 | 7.658 | 169 |
|  |  |  |  |  |  |  |  |
| 10 |  |  |  |  |  |  |  |
| WT vs. FeSOD-A-/-/+c1 | -52.57 | -65.95 to -39.18 | Yes | **** | <0.0001 | 8.882 | 169 |
| WT vs. FeSOD-A-/-/+c2 | -54.54 | -67.93 to -41.16 | Yes | **** | <0.0001 | 9.216 | 169 |
|  |  |  |  |  |  |  |  |
| 12.5 |  |  |  |  |  |  |  |
| WT vs. FeSOD-A-/-/+c1 | -52.17 | -65.55 to -38.78 | Yes | **** | <0.0001 | 8.814 | 169 |
| WT vs. FeSOD-A-/-/+c2 | -57.45 | -70.83 to -44.06 | Yes | **** | <0.0001 | 9.706 | 169 |
|  |  |  |  |  |  |  |  |
| 15 |  |  |  |  |  |  |  |
| WT vs. FeSOD-A-/-/+c1 | -53.63 | -67.02 to -40.25 | Yes | **** | <0.0001 | 9.062 | 169 |
| WT vs. FeSOD-A-/-/+c2 | -57.76 | -71.15 to -44.38 | Yes | **** | <0.0001 | 9.76 | 169 |
|  |  |  |  |  |  |  |  |
| 20 |  |  |  |  |  |  |  |
| WT vs. FeSOD-A-/-/+c1 | -21.87 | -33.84 to -9.893 | Yes | *** | 0.0001 | 4.13 | 169 |
| WT vs. FeSOD-A-/-/+c2 | -39.17 | -51.51 to -26.83 | Yes | **** | <0.0001 | 7.178 | 169 |
|  |  |  |  |  |  |  |  |
| 22.5 |  |  |  |  |  |  |  |
| WT vs. FeSOD-A-/-/+c1 | -4.766 | -25.50 to 15.97 | No | ns | >0.9999 | 0.5198 | 169 |
| WT vs. FeSOD-A-/-/+c2 | -22.59 | -45.78 to 0.5911 | No | ns | 0.0578 | 2.204 | 169 |
|  |  |  |  |  |  |  |  |
| 25 |  |  |  |  |  |  |  |
| WT vs. FeSOD-A-/-/+c1 | 2.699 | -18.04 to 23.44 | No | ns | >0.9999 | 0.2944 | 169 |
| WT vs. FeSOD-A-/-/+c2 | -10.05 | -30.79 to 10.68 | No | ns | 0.5487 | 1.097 | 169 |

| **AMB** |  |  |  |  |  |  |  |
| --- | --- | --- | --- | --- | --- | --- | --- |
|  |  |  |  |  |  |  |  |
| **Bonferroni's multiple comparisons test** | **Mean Diff.** | **95.00% CI of diff.** | **Significant?** | **Summary** | **Adjusted P Value** | **t** | **DF** |
|  |  |  |  |  |  |  |  |
| 0 |  |  |  |  |  |  |  |
| WT vs. FeSOD-A-/-/+c1 | 0 | -13.26 to 13.26 | No | ns | >0.9999 | 0 | 155 |
| WT vs. FeSOD-A-/-/+c2 | 0 | -13.26 to 13.26 | No | ns | >0.9999 | 0 | 155 |
|  |  |  |  |  |  |  |  |
| 0.025 |  |  |  |  |  |  |  |
| WT vs. FeSOD-A-/-/+c1 | -0.1984 | -7.857 to 7.460 | No | ns | >0.9999 | 0.05865 | 155 |
| WT vs. FeSOD-A-/-/+c2 | -2.137 | -9.795 to 5.521 | No | ns | >0.9999 | 0.6315 | 155 |
|  |  |  |  |  |  |  |  |
| 0.05 |  |  |  |  |  |  |  |
| WT vs. FeSOD-A-/-/+c1 | -1.418 | -9.076 to 6.240 | No | ns | >0.9999 | 0.4191 | 155 |
| WT vs. FeSOD-A-/-/+c2 | 1.958 | -5.700 to 9.616 | No | ns | >0.9999 | 0.5786 | 155 |
|  |  |  |  |  |  |  |  |
| 0.075 |  |  |  |  |  |  |  |
| WT vs. FeSOD-A-/-/+c1 | -1.528 | -9.186 to 6.130 | No | ns | >0.9999 | 0.4516 | 155 |
| WT vs. FeSOD-A-/-/+c2 | -1.679 | -9.337 to 5.979 | No | ns | >0.9999 | 0.4962 | 155 |
|  |  |  |  |  |  |  |  |
| 0.1 |  |  |  |  |  |  |  |
| WT vs. FeSOD-A-/-/+c1 | 14.64 | 6.746 to 22.53 | Yes | **** | <0.0001 | 4.198 | 155 |
| WT vs. FeSOD-A-/-/+c2 | 3.055 | -4.839 to 10.95 | No | ns | 0.7648 | 0.876 | 155 |
|  |  |  |  |  |  |  |  |
| 0.125 |  |  |  |  |  |  |  |
| WT vs. FeSOD-A-/-/+c1 | -1.22 | -10.28 to 7.841 | No | ns | >0.9999 | 0.3048 | 155 |
| WT vs. FeSOD-A-/-/+c2 | -10.55 | -19.61 to -1.489 | Yes | * | 0.0185 | 2.635 | 155 |
|  |  |  |  |  |  |  |  |
| 0.15 |  |  |  |  |  |  |  |
| WT vs. FeSOD-A-/-/+c1 | 6.088 | -2.974 to 15.15 | No | ns | 0.2608 | 1.521 | 155 |
| WT vs. FeSOD-A-/-/+c2 | -3.717 | -12.78 to 5.345 | No | ns | 0.7093 | 0.9284 | 155 |
|  |  |  |  |  |  |  |  |
| 0.175 |  |  |  |  |  |  |  |
| WT vs. FeSOD-A-/-/+c1 | -2.316 | -12.15 to 7.522 | No | ns | >0.9999 | 0.5328 | 155 |
| WT vs. FeSOD-A-/-/+c2 | -3.584 | -13.42 to 6.253 | No | ns | 0.8216 | 0.8247 | 155 |

| **Menadione** |  |  |  |  |  |  |  |
| --- | --- | --- | --- | --- | --- | --- | --- |
|  |  |  |  |  |  |  |  |
| **Bonferroni's multiple comparisons test** | **Mean Diff.** | **95.00% CI of diff.** | **Significant?** | **Summary** | **Adjusted P Value** | **t** | **DF** |
|  |  |  |  |  |  |  |  |
| 0 |  |  |  |  |  |  |  |
| WT vs. FeSOD-A-/-/+c1 | 0 | -10.26 to 10.26 | No | ns | >0.9999 | 0 | 135 |
| WT vs. FeSOD-A-/-/+c2 | 0 | -10.26 to 10.26 | No | ns | >0.9999 | 0 | 135 |
|  |  |  |  |  |  |  |  |
| 0.75 |  |  |  |  |  |  |  |
| WT vs. FeSOD-A-/-/+c1 | 18.16 | 12.06 to 24.27 | Yes | **** | <0.0001 | 6.743 | 135 |
| WT vs. FeSOD-A-/-/+c2 | 35.09 | 28.47 to 41.71 | Yes | **** | <0.0001 | 12.01 | 135 |
|  |  |  |  |  |  |  |  |
| 1 |  |  |  |  |  |  |  |
| WT vs. FeSOD-A-/-/+c1 | 23 | 17.07 to 28.92 | Yes | **** | <0.0001 | 8.8 | 135 |
| WT vs. FeSOD-A-/-/+c2 | 36.23 | 29.61 to 42.85 | Yes | **** | <0.0001 | 12.4 | 135 |
|  |  |  |  |  |  |  |  |
| 1.25 |  |  |  |  |  |  |  |
| WT vs. FeSOD-A-/-/+c1 | 21.1 | 14.11 to 28.09 | Yes | **** | <0.0001 | 6.843 | 135 |
| WT vs. FeSOD-A-/-/+c2 | 29.66 | 22.41 to 36.92 | Yes | **** | <0.0001 | 9.269 | 135 |
|  |  |  |  |  |  |  |  |
| 1.5 |  |  |  |  |  |  |  |
| WT vs. FeSOD-A-/-/+c1 | 24.47 | 16.09 to 32.84 | Yes | **** | <0.0001 | 6.621 | 135 |
| WT vs. FeSOD-A-/-/+c2 | 31.98 | 23.09 to 40.86 | Yes | **** | <0.0001 | 8.158 | 135 |
|  |  |  |  |  |  |  |  |
| 1.75 |  |  |  |  |  |  |  |
| WT vs. FeSOD-A-/-/+c1 | 9.569 | 2.947 to 16.19 | Yes | ** | 0.0027 | 3.275 | 135 |
| WT vs. FeSOD-A-/-/+c2 | 12.44 | 5.185 to 19.69 | Yes | *** | 0.0003 | 3.887 | 135 |
|  |  |  |  |  |  |  |  |
| 2.0 |  |  |  |  |  |  |  |
| WT vs. FeSOD-A-/-/+c1 | 12.17 | 5.549 to 18.79 | Yes | *** | 0.0001 | 4.166 | 135 |
| WT vs. FeSOD-A-/-/+c2 | 11.44 | 4.185 to 18.69 | Yes | *** | 0.001 | 3.575 | 135 |
|  |  |  |  |  |  |  |  |
| 2.5 |  |  |  |  |  |  |  |
| WT vs. FeSOD-A-/-/+c1 | 7.409 | 1.486 to 13.33 | Yes | * | 0.0106 | 2.835 | 135 |
| WT vs. FeSOD-A-/-/+c2 | 5.924 | -0.6980 to 12.55 | No | ns | 0.0891 | 2.028 | 135 |
|  |  |  |  |  |  |  |  |
